# Deep Radiogenomics Sequencing for Breast Tumor Gene-Phenotype Decoding Using Dynamic Contrast Magnetic Resonance Imaging

**DOI:** 10.1007/s11307-025-01981-x

**Published:** 2025-01-15

**Authors:** Isaac Shiri, Yazdan Salimi, Pooya Mohammadi Kazaj, Sara Bagherieh, Mehdi Amini, Abdollah Saberi Manesh, Habib Zaidi

**Affiliations:** 1 Division of Nuclear Medicine and Molecular Imaging, Geneva University Hospital, CH-1211 Geneva, Switzerland; 2 K N Toosi University of Technology, Tehran, Tehran, Iran; 3 School of Medicine, Isfahan University of Medical Sciences, Isfahan, Iran; 4 Department of Nuclear Medicine and Molecular Imaging, University of Groningen, University Medical Center Groningen, Groningen, Netherlands; 5 Department of Nuclear Medicine, University of Southern Denmark, Odense, Denmark; 6 University Research and Innovation Center, Óbuda University, Budapest, Hungary

**Keywords:** Radiogenomics, Breast, MRI, Deep learning, Progesterone receptors, Estrogen receptors, HER2

## Abstract

**Purpose:**

We aim to perform radiogenomic profiling of breast cancer tumors using dynamic contrast magnetic resonance imaging (MRI) for the estrogen receptor (ER), progesterone receptor (PR), and human epidermal growth factor receptor 2 (HER2) genes.

**Methods:**

The dataset used in the current study consists of imaging data of 922 biopsy-confirmed invasive breast cancer patients with ER, PR, and HER2 gene mutation status. Breast MR images, including a T1-weighted pre-contrast sequence and three post-contrast sequences, were enrolled for analysis. All images were corrected using N4 bias correction algorithms. Based on all images and tumor masks, a bounding box of 128 × 128 × 68 was chosen to include all tumor regions. All networks were implemented in 3D fashion with input sizes of 128 × 128 × 68, and four images were input to each network for multi-channel analysis. Data were randomly split into train/validation (80%) and test set (20%) with stratification in class (patient-wise), and all metrics were reported in 20% of the untouched test dataset.

**Results:**

For ER prediction, SEResNet50 achieved an AUC mean of 0.695 (CI95%: 0.610–0.775), a sensitivity of 0.564, and a specificity of 0.787. For PR prediction, ResNet34 achieved an AUC mean of 0.658 (95% CI: 0.573–0.741), a sensitivity of 0.593, and a specificity of 0.734. For HER2 prediction, SEResNext101 achieved an AUC mean of 0.698 (95% CI: 0.560–0.822), a sensitivity of 0.750, and a specificity of 0.625.

**Conclusion:**

The current study demonstrated the feasibility of imaging gene-phenotype decoding in breast tumors using MR images and deep learning algorithms with moderate performance.

## Introduction

With millions of annual cases, breast cancer has become an escalating source of morbidity and one of the leading causes of cancer mortality among women [1, 2]. Different studies reported that although the incidence rates continue to surge, the worldwide survival rates have improved, leading to a growing number of breast cancer survivors [1–3]. Some contributing factors include screening strategies, higher-precision diagnostic methods, and novel therapeutic strategies [3–7].

Genetic testing and breast cancer gene decoding gained popularity following the discovery of BRCA1 and BRCA2 genes as contributors to hereditary breast and ovarian cancers [8]. It is now well established that in breast cancer cases, genetic alterations and somatic mutations play a crucial role in determining tumors’ biological and clinical characteristics, including response to treatment [9]. For example, progesterone receptors (PR), estrogen receptors (ER), and human epidermal growth factor receptor-2 (HER2) are among the most investigated receptors whose expression on the surface of breast cells is regulated by genetic composition [10, 11]. The development of receptor-specific biomarkers and targeted therapies has advanced early cancer diagnosis and improved the prognosis for breast cancer survivors [12–14].

Artificial intelligence-based algorithms, such as deep learning algorithms, have become widely popular in medicine due to their ability to identify different patterns [15]. Artificial intelligence-based models are utilized in various aspects of medical imaging, including cancer diagnosis and prognosis, offering accurate results through radiomic and radiogenomic analyses [16–22]. Radiomics extracts features that quantify the size, shape, and texture of the region of interest, offering comprehensive and personalized tumor characterization [23]. Radiogenomics aims to determine gene status using imaging phenotypes, which can be achieved through radiomics analysis or end-to-end deep learning algorithms [24].

Previous studies attempted to identify associations between radiological molecular and genomics features [25, 26]. A machine-learning algorithm was designed to predict the link between imaging features and gene expressions [25], achieving a moderate performance for ER-positive breast cancer. Another study found no association between HER2 expression and imaging features [26]. Previous studies predominantly relied on radiomic features extracted from the gross tumor volume excluding the peritumoral region, which is potentially important for providing a holistic overview of the tumor. Moreover, most radiomics and deep learning-based analyses were conducted on small datasets and typically used a single machine/deep learning model. The current study attempts to address these limitations by applying a comprehensive deep learning-based algorithm to a large breast cancer dataset, aiming to achieve a more realistic assessment of deep learning model performance in predicting gene status in breast cancer patients. In this study, we explore the applicability of deep radiogenomics sequencing for breast tumor gene-phenotype decoding. We aimed to perform radiogenomic profiling of breast cancer patients using their dynamic contrast-enhanced MR images and deep learning algorithms.

## Materials and Methods

### Image Acquisition

The dataset used in the current study was acquired from The Cancer Imaging Archive (TCIA) and consisted of demographic, clinical, pathology, treatment, outcomes, and genomic data of 922 biopsy-confirmed invasive breast cancer patients over a decade, along with their de-identified pre-operative dynamic contrast-enhanced (DCE)-MRI images [25, 27, 28].

Gene status determination was performed by immunohistochemistry (IHC) analysis [25, 27, 28]. Regarding ER and PR status determination, an Allred score of 3 and above was considered ER-positive and PR-positive, whereas for HER2 status determination, a HER2 score of 3, or 2 with an additional condition associated with an amplification of HER2 gene, assessed using Fluorescence in Situ Hybridization (FISH), was necessary for the tumor to be considered HER2-positive [25, 27, 28]. In terms of imaging data, (i) a non-fat saturated T1-weighted, (ii) a fat-saturated gradient echo T1-weighted pre-contrast, and (iii) four post-contrast T1-weighted axial breast MRIs in a prone position were acquired on 1.5T or 3T scanners [25, 27, 28]. The MR imaging parameters were as follows: slice thickness ranged from 1.04 to 2.5 mm, repetition time (TR) ranged from 3.54 to 7.39 ms, echo time (TE) ranged from 1.25 to 2.76 ms, and flip angle ranged from 7 to 12 degrees [25, 27, 28]. The acquisition matrix ranged from 320 × 320 to 448 × 448, with a field-of-view between 250 and 480 mm. Scans were performed using a 1.5T (3 GE and 1 Siemens scanners) or 3T (3 GE and 3 Siemens scanners) scanner [25, 27, 28]. More detailed information about the dataset can be found in [25, 27, 28].

### Preprocessing

Breast MR images were used in this study, including a T1-weighted pre-contrast sequence and three post-contrast sequences. All images were corrected using N4 bias correction algorithms with default parameters built in the ITK library [29, 30]. The tumor region was delineated using three-dimensional bounding boxes based on tumor masks provided in the original dataset [25]. All images were cropped to the region, including tumors, using the provided tumor mask. Based on all images, a bounding box of 128 × 128 × 68 was chosen to include all tumor regions, and no further image resizing was performed to preserve the original image resolution. Image intensity was normalized to [0–1] for further evaluation using the min–max approach.

### Deep Learning Algorithm

**Fig. 1:**
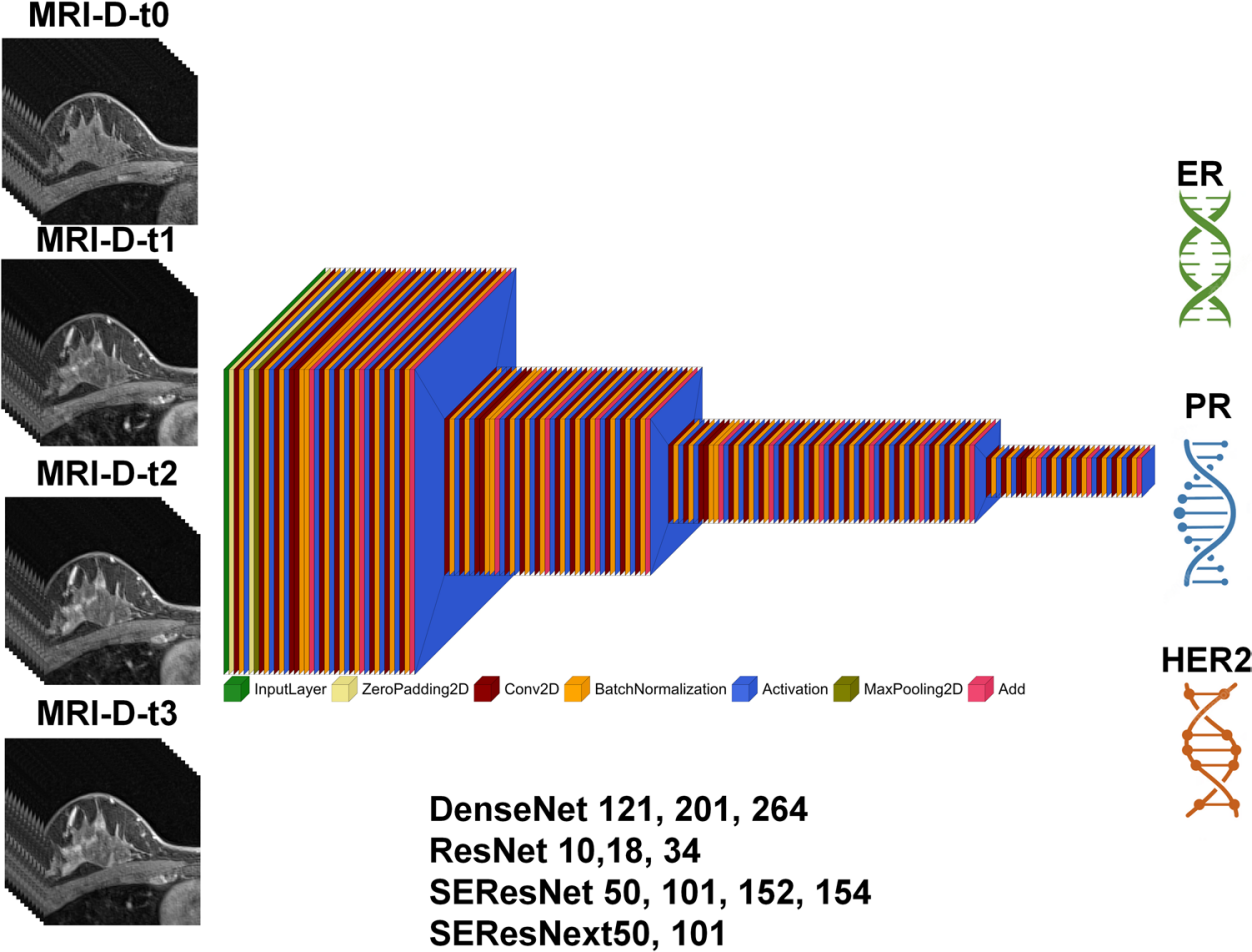
Diagram of the current study including four input channel MR images along with networks and targets

We implemented various 3D deep learning algorithms for gene status prediction, including DenseNet121, DenseNet201, DenseNet264, ResNet10, ResNet18, ResNet34, SEResNet50, SEResNet101, SEResNet152, SENet154, SEResNext50, and SEResNext101. These networks represent diverse generations of different architectures, incorporating dense connections, residual blocks, squeeze-and-excitation modules, and squeeze-and-excitation residual blocks with varying numbers of layers. The inclusion of these models allows for a comprehensive evaluation of their distinct strengths in different breast gene classifications. All networks were implemented in 3D approaches with input sizes of 128 × 128 × 68, and four images (T1-weighted pre-contrast and three post-contrast images) were input to each network for multi-channel analysis. A maximum batch size of 4 was chosen for training (due to limited GPU memory), and all networks were trained using 100 epochs. The training was performed using the Adam optimizer, an initial learning rate of 10^–5^, and a decay of 0.05. A schematic of the network architecture is presented in Fig. 1.

For different genes, the dataset exhibited class imbalances (positive/negative %, classes), with proportions of 74/26% for ER, 65/35% for PR, and 18/82% for HER2. This was addressed by applying data augmentation techniques, including zooming, intensity adjustments, and rotation, to improve model robustness and reduce overfitting by increasing the diversity of training samples. All models were developed on PyTorch [31] and MONAI [32] libraries using a single RTX 2080 Ti GPU. Model parameter and hyperparameter (best epoch based on validation) optimization was conducted during training to fine-tune each model, with the batch size constrained by available GPU resources.

### Evaluation Strategy

All data were randomly split into train/validation (80%) and test set (20%) with stratification in class (patients-wise), and all metrics were reported in 20% of the untouched test dataset. Different metrics, including precision, sensitivity, specificity, F1, Accuracy, Balanced Accuracy, and area under the receiver operating characteristic curve (AUC), were reported for the hold-out test sets. In addition, all trained models were compared using the DeLong test for statistical differences in AUC. The significance level was considered at a level of 0.05.

## Results

### ER Classification Metrics

**Fig. 2:**
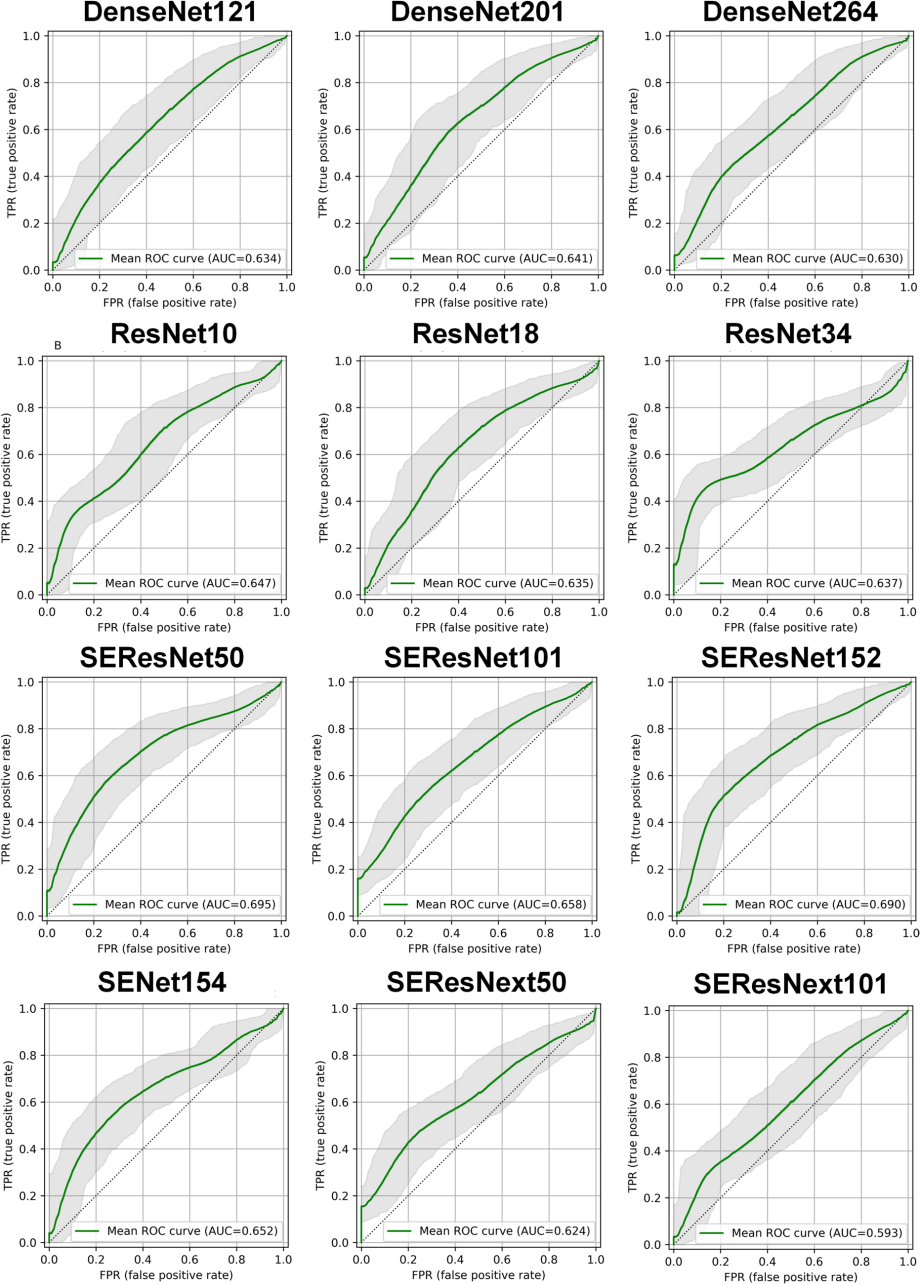
ROC curves of different networks for ER prediction (Minor discrepancies between the table and plot AUC values are attributable to the 10,000 bootstrapping iterations employed during plotting and also different libraries used for the metrics calculations)

Table 1 summarizes classification metrics for ER gene prediction. Among the 12 classification networks that were used, ResNet10 had the highest accuracy (0.665), followed by DenseNet121 (0.643) and DenseNet201 (0.637). In terms of AUC, the highest rates were achieved with SEResNet50, SEResNet152, and SEResNet101 with AUCs of 0.695 (CI95%: 0.61 – 0.775), 0.691 (CI95%: 0.601 – 0.776), and 0.659 (CI95%: 0.57 – 0.744), respectively. Statistically significant difference was observed between SEResNext101 (AUC = 0.592) and SEResNet50 (AUC = 0.695). Figure 2 depicts the ROC curve of different models for ER prediction.

**Table 1:** Summary of classification metrics for different networks in ER Gene prediction. Minor discrepancies between the plotted AUC values are attributable to the 10,000 bootstrapping iterations employed during plotting a single curve also different libraries used for the metrics calculations

Metrics	Accuracy	Balanced Accuracy	Sensitivity	Specificity	F1	AUC (mean)	AUC (CI95%)
DenseNet121	0.643	0.614	0.674	0.553	0.737	0.635	(0.542, 0.727)
DenseNet201	0.637	0.645	0.630	0.660	0.720	0.642	(0.548, 0.734)
DenseNet264	0.516	0.612	0.415	0.809	0.560	0.629	(0.535, 0.720)
ResNet10	0.665	0.628	0.704	0.553	0.757	0.646	(0.552, 0.732)
ResNet18	0.577	0.632	0.519	0.745	0.645	0.634	(0.540, 0.726)
ResNet34	0.577	0.673	0.474	0.872	0.624	0.637	(0.553, 0.716)
SEResNet50	0.621	0.675	0.563	0.787	0.688	0.695	(0.610, 0.775)
SEResNet101	0.615	0.637	0.593	0.681	0.696	0.659	(0.570, 0.744)
SEResNet152	0.621	0.668	0.570	0.766	0.691	0.691	(0.601, 0.776)
SENet154	0.555	0.651	0.452	0.851	0.601	0.652	(0.562, 0.736)
SEResNext50	0.549	0.627	0.467	0.787	0.606	0.622	(0.535, 0.708)
SEResNext101	0.467	0.606	0.319	0.894	0.470	0.592	(0.498, 0.684)

### PR Classification Metrics

**Fig. 3:**
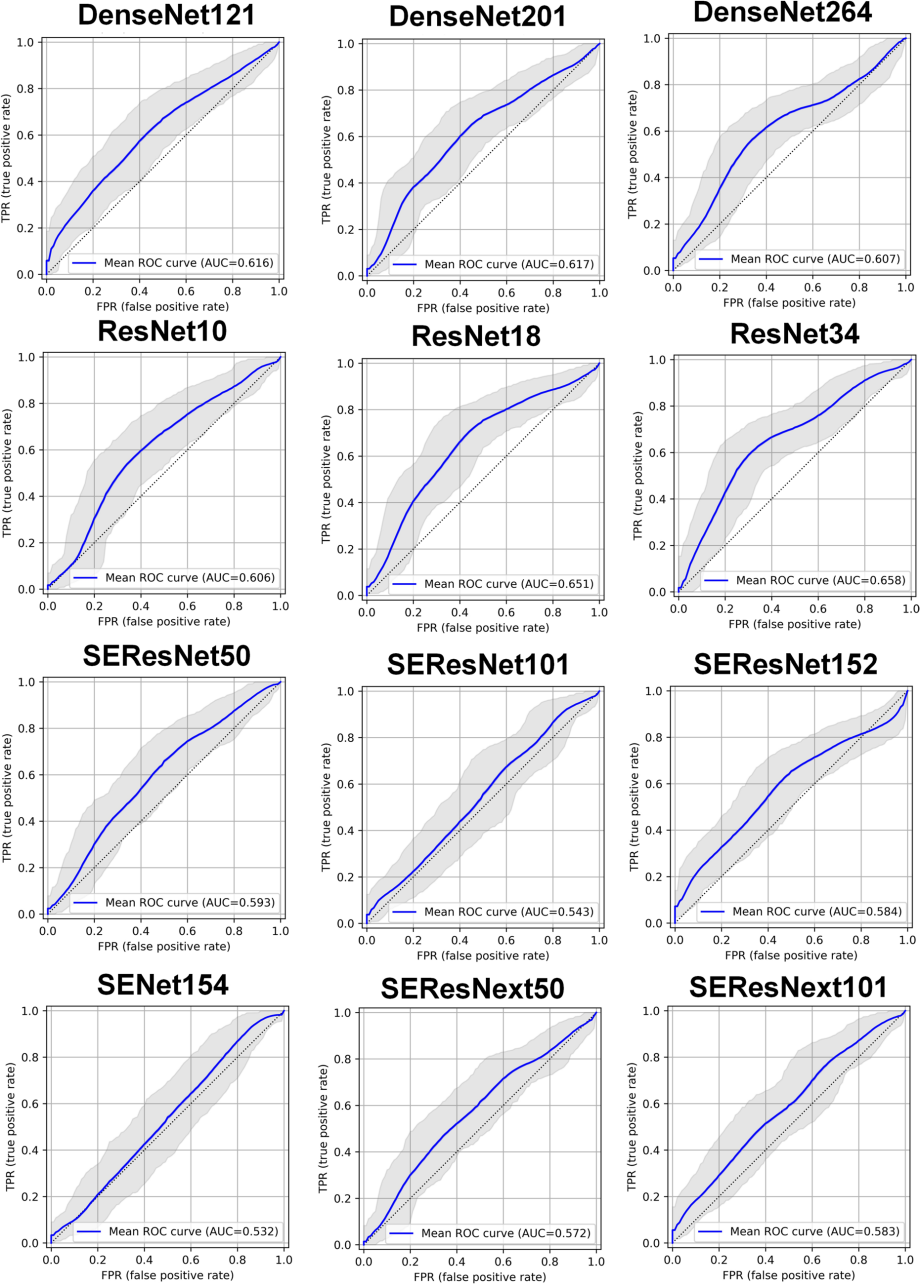
ROC curves of different networks for PR prediction (Minor discrepancies between the table and plot AUC values are attributable to the 10,000 bootstrapping iterations employed during plotting also different libraries used for the metrics calculations)

Table 2 summarizes classification metrics for PR gene prediction. Among the 12 utilized classification networks, ResNet18 had the highest accuracy (0.665). Regarding AUC, the highest performance was observed using ResNet34, ResNet18, and DenseNet201 with AUCs of 0.658 (95% CI: 0.573–0.741), 0.650 (95% CI: 0.563–0.731), and 0.619 (95% CI: 0.531–0.701), respectively. Considering all the different metrics, ResNet18 outperformed the other networks. Figure 3 depicts the ROC curves of different models for PR prediction.

**Table 2:** Summary of classification metrics for different networks in PR gene prediction. Minor discrepancies between the plotted AUC values are attributable to the 10,000 bootstrapping iterations employed during plotting a single curve also different libraries used for the metrics calculations

Metrics	Accuracy	Balanced Accuracy	Sensitivity	Specificity	F1	AUC (mean)	AUC (CI95%)
DenseNet121	0.604	0.598	0.619	0.578	0.670	0.616	(0.529, 0.699)
DenseNet201	0.643	0.624	0.686	0.563	0.714	0.619	(0.531, 0.701)
DenseNet264	0.604	0.627	0.551	0.703	0.644	0.606	(0.518, 0.689)
ResNet10	0.582	0.614	0.508	0.719	0.612	0.605	(0.516, 0.692)
ResNet18	0.665	0.649	0.703	0.594	0.731	0.650	(0.563, 0.731)
ResNet34	0.643	0.664	0.593	0.734	0.683	0.658	(0.573, 0.741)
SEResNet50	0.615	0.589	0.678	0.500	0.696	0.593	(0.501, 0.677)
SEResNet101	0.599	0.562	0.686	0.438	0.689	0.543	(0.453, 0.633)
SEResNet152	0.599	0.591	0.619	0.563	0.667	0.584	(0.498, 0.667)
SENet154	0.648	0.550	0.881	0.219	0.765	0.530	(0.439, 0.620)
SEResNext50	0.560	0.586	0.500	0.672	0.596	0.576	(0.487, 0.661)
SEResNext101	0.566	0.576	0.542	0.609	0.618	0.584	(0.496, 0.668)

### HER2 Classification Metrics

**Fig. 4:**
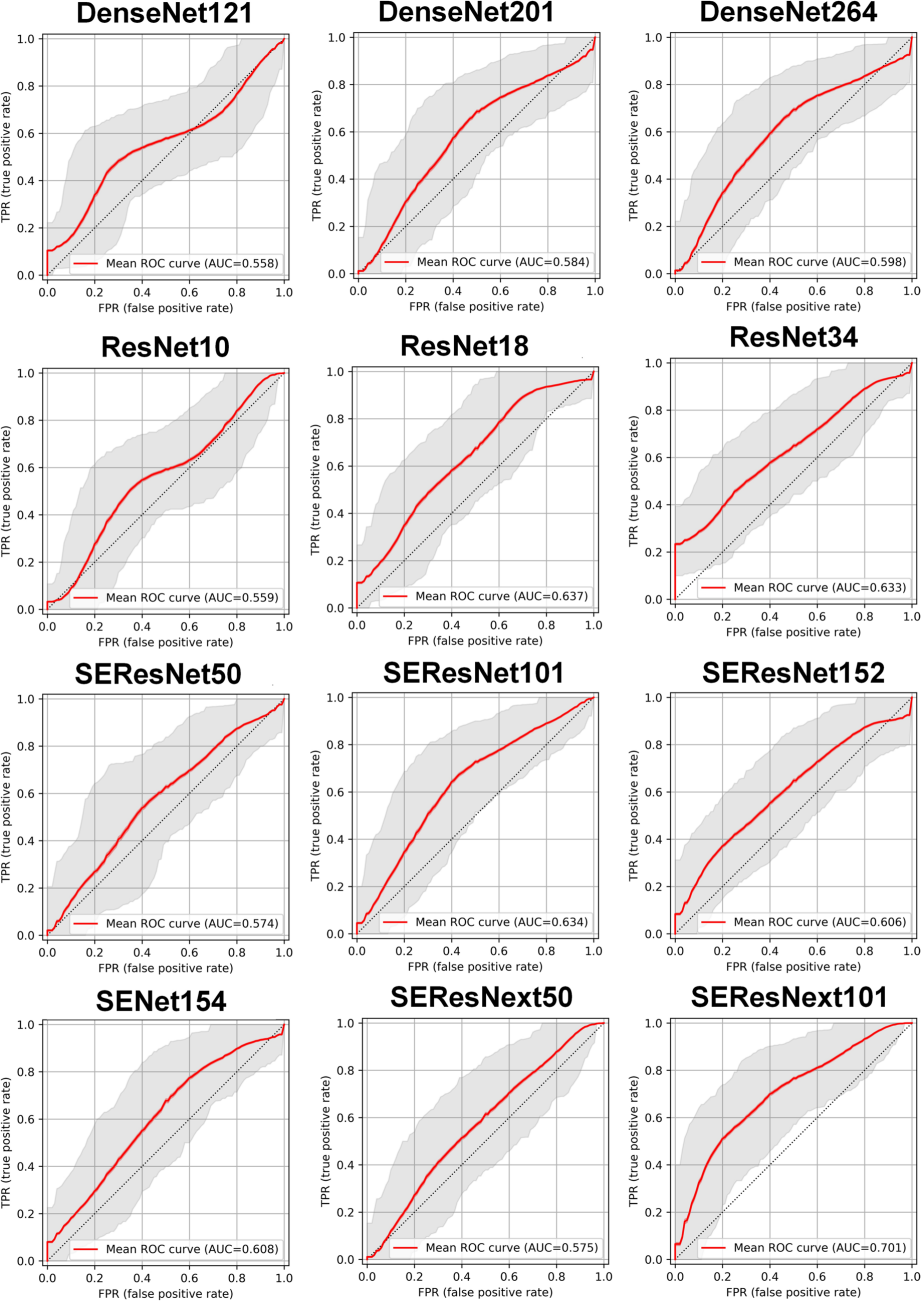
ROC curves of different networks for HER2 prediction (Minor discrepancies between the table and plot AUC values are attributable to the 10,000 bootstrapping iterations employed during plotting also different libraries used for the metrics calculations)

Table 3 summarizes classification metrics for HER2 gene prediction. Among the 12 classification networks that were deployed, SEResNext101 had the highest accuracy (0.688), followed by DenseNet121 (0.641), ResNet18 (0.641), and SEResNet101 (0.641). Considering AUC, the highest performance was obtained with SEResNext101, ResNet18, and SEResNet101 with AUCs of 0.698 (95% CI: 0.56–0.822), 0.637 (95% CI: 0.495–0.772), and 0.634 (95% CI: 0.491–0.767), respectively. The SEResNet101 outperformed other algorithms when considering all different metrics. Figure 4 depicts the ROC curve of different models for HER2 prediction.

**Table 3:** Summary of classification metrics for different networks in HER2 Gene prediction. Minor discrepancies between the plotted AUC values are attributable to the 10,000 bootstrapping iterations employed during plotting a single curve also different libraries used for the metrics calculations

Metrics	Accuracy	Balanced Accuracy	Sensitivity	Specificity	F1	AUC (mean)	AUC (CI95%)
DenseNet121	0.641	0.641	0.500	0.781	0.582	0.559	(0.411, 0.702)
DenseNet201	0.625	0.625	0.656	0.594	0.636	0.580	(0.435, 0.721)
DenseNet264	0.625	0.625	0.750	0.500	0.667	0.596	(0.451, 0.739)
ResNet10	0.609	0.609	0.500	0.719	0.561	0.560	(0.414, 0.702)
ResNet18	0.641	0.641	0.938	0.344	0.723	0.637	(0.495, 0.772)
ResNet34	0.625	0.625	0.500	0.750	0.571	0.630	(0.490, 0.764)
SEResNet50	0.625	0.625	0.563	0.688	0.600	0.567	(0.421, 0.707)
SEResNet101	0.641	0.641	0.750	0.531	0.676	0.634	(0.491, 0.767)
SEResNet152	0.594	0.594	0.750	0.438	0.649	0.606	(0.461, 0.746)
SENet154	0.625	0.625	0.781	0.469	0.676	0.604	(0.464, 0.740)
SEResNext50	0.594	0.594	0.813	0.375	0.667	0.580	(0.437, 0.724)
SEResNext101	0.688	0.688	0.750	0.625	0.706	0.698	(0.560, 0.822)

**Fig. 5:**
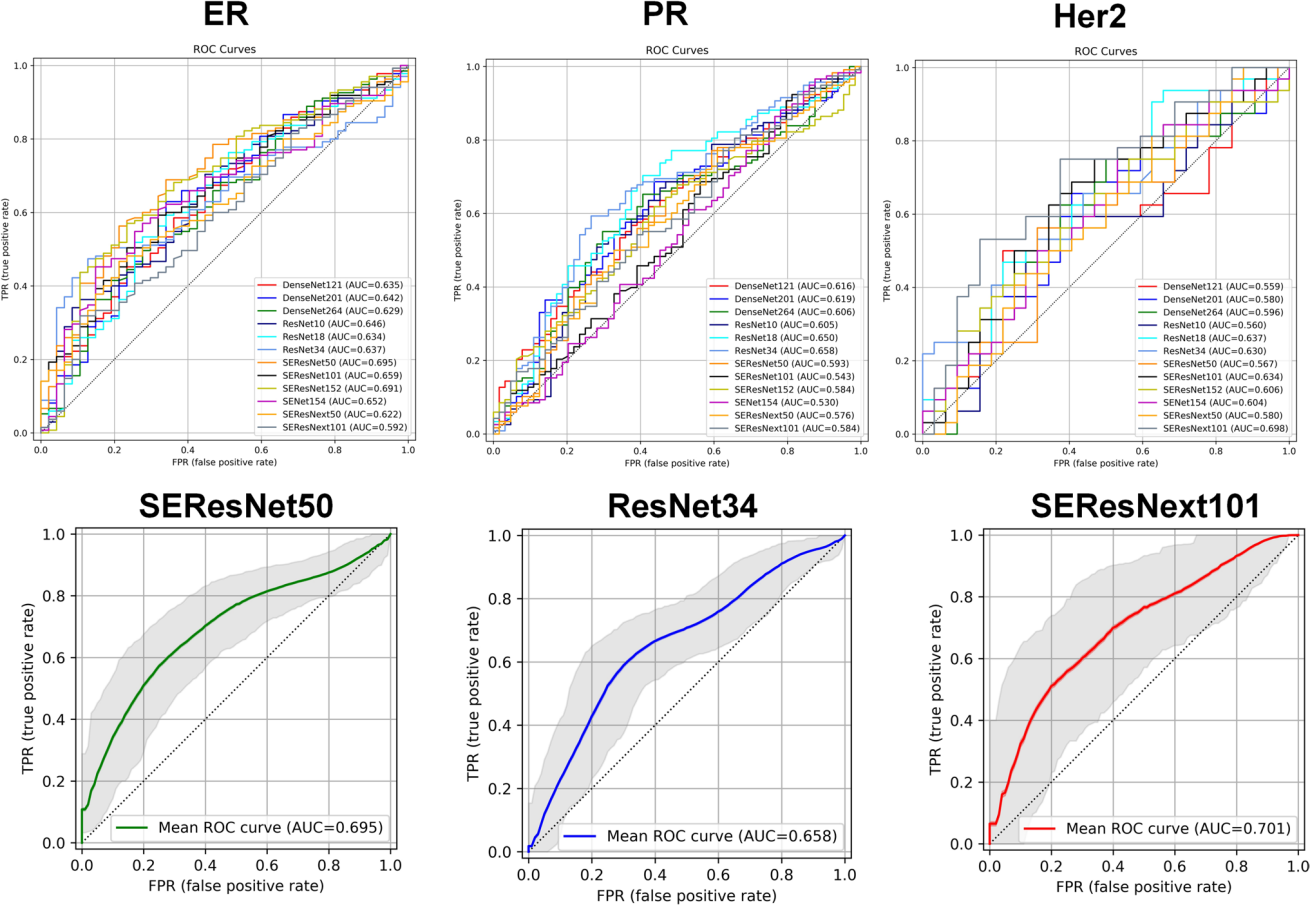
ROC curves of different networks for different gene prediction in all networks and best-performing network for each gene (Minor discrepancies between the plotted AUC values are attributable to the 10,000 bootstrapping iterations employed during plotting a single curve also different libraries used for the metrics calculations)

Figure 5 represents the ROC curves of different models in the prediction of genes for better comparison and the best model of each gene prediction. For ER prediction, SEResNet50 achieved an AUC mean of 0.695 (CI95%: 0.610–0.775), a sensitivity of 0.564, and a specificity of 0.787. For PR prediction, ResNet34 achieved an AUC mean of 0.658 (95% CI: 0.573–0.741), a sensitivity of 0.593, and a specificity of 0.734. For HER2 prediction, SEResNext101 achieved an AUC mean of 0.698 (95% CI: 0.560–0.822), a sensitivity of 0.750, and a specificity of 0.625.

## Discussion

Identifying the receptors of cancer cells through genetic analysis is essential for accurate diagnosis and guiding therapy toward precision medicine in cancer patients [33, 34]. For instance, trastuzumab, commonly known as Herceptin, is an FDA-approved monoclonal antibody used to treat HER2-positive cancers, including breast cancer [35, 36]. Different studies have provided evidence of the better outcome of adjuvant therapy in patients with early HER2-positive breast cancer [11, 36–39]. The discovery of trastuzumab was made possible by advancements in genetic analysis and gene phenotyping of cancer cells [40, 41]. Similarly, estrogen and progesterone receptor blockers are used to treat ER-positive and PR-positive tumors, respectively [42]. The use of receptor-specific agents has been demonstrated in numerous studies to be effective, leading to improvements in both overall survival and disease-free survival rates [36]. The results of this study showed that deep radiogenomic sequencing can decode the gene-phenotype characteristics of breast lesions from MR images, achieving a moderate AUC of approximately 0.70.

Various studies have employed artificial intelligence algorithms to enhance the diagnosis and prognosis of cancer patients [43, 44]. Wang et al. [45] applied radiomics to predict the prognosis of locally advanced breast cancer (LABC) patients after neoadjuvant chemotherapy and radiotherapy. Their model classified patients into distinct radiomics score groups with unique gene expression patterns and immunophenotype compositions [45]. A study involving ultrasonography breast images from 187 women, including 68 malignant and 119 benign cases, reported classification accuracy of 87%, sensitivity of 77%, and specificity of 92%. They suggested that neighboring regions, such as the perfusion region and marginal region, besides the tumoral region, can be of use in diagnosing breast tumors [46]. Another study has reported deep learning-based radiomic analysis of breast lesions to classify benign and malignant tumors, including peritumoral tissue [47]. The deep learning-based analysis in this investigation enabled a fully automatic diagnostic process, from segmentation to detection and classification. Zhou et al. [47] assessed DCE-MR images of 133 patients with radiomics and deep learning. The diagnostic accuracy of ResNet50 in the testing dataset reached 89%, demonstrating the importance of including peritumoral regions in radiomics analysis to achieve more robust results [47]. However, considering the relatively small sample size, such high indices are potentially liable to be biased and require further evaluation on larger, more diverse sample sizes.

Radiogenomics aims to provide a more comprehensive characterization of tumors by deciphering the cancer genotype from radiological images [48]. Compared to genomic data alone, combining the radiomic feature and genomic information could potentially provide several advantages [48]. First, radiomics could potentially allow the analysis of tumor characteristics more comprehensively, which lowers the risk of selection bias and increases the generalizability of the findings [49]. Second, tumor size, margins, vascularity, and other morphological features have been shown to correlate with the transcriptional activity of multiple genetic pathways [50]. Thus, incorporating these data types improves diagnostic and prognostic accuracy and predictive models. Finally, having patient-specific radiology reports with the genome information enables physicians to personalize their treatment plans to achieve maximum efficacy using non-invasive methods [49, 50].

Various radiogenomic studies focused on breast cancer using artificial intelligence-based algorithms. A multi-parametric MRI radiomics and machine learning study was conducted on 162 patients using DCE T1-weighted images, fat-suppressed T2-weighted images, and apparent diffusion coefficient maps [51]. They reported an AUC of 0.84 and 0.86 in differentiating HER2-positive form HER2-negative cases and HR2-positive/HER2-negative tumors versus other cases, respectively [51]. Saha et al. [25] reported a moderate association between the molecular receptors on cancer cells’ surface and machine learning-based predictions based on imaging features. The reported AUC of ER-positive, PR-positive, and HER2-positive breast cancer detection were 0.65, 0.62, and about 0.50, respectively [25]. Our study was conducted on the same dataset used by Saha et al. [25]. Our results outperformed theirs (0.69 vs. 0.649, 0.658 vs. 0.622, and 0.701 vs. 0.500 for ER, PR, and HER2), which could be due to various reasons, including (i) the deployment of deep learning, leading to a lower level of uncertainty in region of interest selection, segmentation, feature extraction, etc., and (ii) analyzing the whole image instead of exclusively selecting the tumor, including the peripheral regions and neighboring tissues, which could result in improvements in prediction.

In another radiogenomics study, DCE-MRI and RNA sequencing from 47 patients were used to find gene pathways most linked to radiomic features and to see if these links affect the radiological appearance of tumors [52]. They [52] showed that the highest number of significant radiomics associations were with gene pathways concerning the modulation of the immune system and extracellular signaling, with an average of 18.9 and 16 features, respectively [52]. Additionally, the upregulation of the identified signaling pathways was linked to smaller, more spherical tumors with a more heterogeneous texture in contrast-enhanced images [52]. Additionally, the feasibility of deep learning in radiogenomics has been previously assessed. For example, a previous study on 270 breast cancer patients achieved an AUC of 0.65 for distinguishing different molecular subtypes [53]. Zhu et al. [53] explored possible applications of transfer learning in radiogenomics. Their results demonstrated a slight outperformance of transfer learning against training from scratch (0.60 vs. 0.58) [53]. Overall, these studies align with our findings as we hypothesized and demonstrated the connection between tumors’ imaging features and their genetic and biological characteristics.

In this study, we evaluated multiple deep-learning architectures to account for the variability in performance across different tasks. Our results showed that different networks performed best for various gene sets, showing the importance of selecting models based on specific tasks. Translating these findings into clinical practice presents challenges, such as complex data integration, improving model performance, infrastructure needs, deep learning expertise, and prospective validation in diverse patient populations. Differences in imaging protocols, data formats, and preprocessing pipelines create heterogeneity that impacts model performance. These challenges could be potentially addressed using harmonization techniques or synthetic data augmentation to improve robustness and generalizability. Addressing these issues is key to successfully implementing deep radiogenomic models in routine care.

While our study utilized a heterogeneous dataset, which may improve the model's robustness, we did not directly assess its sensitivity to different imaging protocols. Future studies should investigate the impact of varying imaging parameters on model performance to ensure broader clinical applicability. Although we used a large open-access dataset and applied standardized deep learning algorithms during image processing and model development and evaluation to prevent potential data leakage and ensure realistic, generalizable results, the model's performance was not evaluated in an external validation setting. As a result, further testing is needed to assess the model's performance in real-world scenarios thoroughly using external validation datasets. Moreover, using a public dataset resembles a double-edged sword in machine learning studies. On the one hand, it provides an opportunity to be used by different researchers for model development; on the other hand, the more they are used, the more they pose the risk of overfitting some artificial intelligence-based models on the data. Although overfitted models still offer reasonable predictions, their generalizability to real-world settings suffers dramatically and requires further evaluation [54]. Future research should focus on using larger, multicenter datasets to evaluate models across diverse populations, ensuring more robust and generalizable performance. Additionally, exploring newer deep-learning architectures could further enhance model accuracy. Future studies could incorporate explainable AI techniques, such as saliency maps and Grad-CAM (Gradient-weighted Class Activation Mapping), to enhance model interpretability for clinical decision-making. These approaches could provide visual explanations by highlighting the most relevant regions in the images, which could potentially offer greater transparency in clinical settings. Finally, models should be prospectively evaluated in real-world clinical settings to assess their practical utility and performance.

## Conclusion

The current study demonstrated the feasibility of imaging gene-phenotype decoding in breast cancer using MR images. Furthermore, we compared different neural network architectures for ER, PR, and HER2 gene prediction in these patients using MRI images and determined the optimum network for the prediction of these genes. Overall, we demonstrated that a deep radiogenomic model could potentially decode breast cancer gene-phenotype with moderate performance. Further research is needed to better understand the limitations and strengths of deep radiogenomic models when applied in clinical settings.
